# Assistance Robotics and Sensors

**DOI:** 10.3390/s23094286

**Published:** 2023-04-26

**Authors:** Santiago T. Puente, Fernando Torres

**Affiliations:** AUROVA Group, Department of Physics, System Engineering and Signal Theory, University of Alicante, 03690 Alicante, Spain; fernando.torres@ua.es

In recent years, the exploitation of assistive robotics has experienced significant growth, mostly based on the development of sensor and processing technologies with the increasing interest in improving the interactions between robots and humans and making them more natural. Robots are required to assist humans in many industries, in the manufacturing workspace, in the rehabilitation process, as well as in the medical environment. Robots are used to achieve ambient assisted living and to help older adults. Furthermore, assistive robots are used in security, search or rescue operations, and in interactions with humans with infectious diseases.

This Special Issue is focused on breakthrough developments in the field of assistive robotics, including current scientific progress in machine learning, deep learning, reinforcement learning, and imitation learning to enable assistive robots to help humans in any environment, as well as any supportive sensorial system that facilitates the interaction between humans and robots at home or in the industrial environment. In addition to the aforementioned environments, methods and algorithms that combine sensors to enable assistive robots can be considered. This Special Issue covers innovative solutions in these fields.

The Special Issue has collected eight outstanding papers covering different aspects of assistance robotics and sensors. The selected contributions cover several main topics related to assistance robotics, from exoskeletons, human–robot interfaces, surgical robots, and so on. In the following, a brief summary of the scope and main contributions of each of these papers is provided as a teaser for the interested reader.

Neuromotor rehabilitation and recovery of upper limb functions are essential to improve the life quality of patients who have suffered injuries or have pathological sequels, where it is desirable to enhance the development of activities of daily living, such as the use of exoskeletons providing support in diagnostic and rehabilitation processes. The use of a soft material exoskeleton prototype for upper limbs rehabilitation presented in [1] demonstrates that the use of soft materials provides satisfactory outcomes in the motion transfer and support to the limb. The uses of Artificial Intelligence-based control in a wearable robotic exoskeleton with autonomous, processing, and safety algorithms embedded in the device is presented in [2]. Additionally, a study of the payload adjustment for an exoskeleton is discussed in [3].

The human–robot interface enables disabled people to control different devices and is another important area that has been addressed in the present Special Issue. In [4], the authors present a low-cost magnetic field control that is placed on the patient’s tongue which provides an interface to control the navigation of a wheelchair. In [5], an interface that uses head-and-eye gaze is presented to teleoperate 6 Degrees of Freedom robot in a Cartesian space.

An assistive device that helps patients to drink is presented in [6], which combines a robot arm with a vision system to recognize the mouth and autonomously navigate a cup to provide contact with the mouth.

In [7], an omnidirectional wheel design control is presented, to provide a system that can navigate in indoor and outdoor environments.

Finally, in [8], the authors present robot-assisted surgery, performing an study of the acoustic and disruptive factors during the surgery operation in order to measure it and increase patient safety.

In summary, there is a huge potential in assistive robotics and sensors; however, there are still many challenges to address before those techniques can become reality for the user. The potential of assistive robotics in medical applications is great, and the challenges are great too. We encourage authors to continue researching and improving in this field.
